# MicroRNA-503 Suppresses Oral Mucosal Fibroblast Differentiation by Regulating RAS/RAF/MEK/ERK Signaling Pathway

**DOI:** 10.3390/biom14101259

**Published:** 2024-10-05

**Authors:** Dada Wen, Huamin Zhang, Yutong Zhou, Ni Jian, Canhua Jiang, Jie Wang

**Affiliations:** 1 Department of Immunology, Xiangya School of Medicine, Central South University, Changsha 410078, China; wendada0122@163.com (D.W.); 216511058@csu.edu.cn (H.Z.); 226511060@csu.edu.cn (Y.Z.); 236511054@csu.edu.cn (N.J.); 2 Department of Oral and Maxillofacial Surgery, Xiangya Hospital, Central South University, Changsha 410078, China; canhuaj@csu.edu.cn

**Keywords:** platelet-derived growth factor-BB, miR-503, primary oral mucosal fibroblasts, serine/threonine protein kinase (RAF), biological behavior

## Abstract

The abnormal proliferation and differentiation of oral mucosal fibroblasts (FBs) is the key to the progression of oral submucosal fibrosis. To clarify the mechanism of platelet-derived growth factor (PDGF-BB)-induced FBs fibrosis in oral mucosa, real-time quantitative polymerase chain reaction and Western blot were used in this study to detect the expression of miR-503 and the expression of p-MEK, p-ERK, miR-503, RAF, smooth actin and type I collagen under different time and concentration stimulation of PDGF-BB. The effects of overexpression of miR-503 or RAF on the proliferation and migration of FBs were detected by cell counting kit 8 and cell scratch assay, respectively. A dual luciferase reporter gene assay was used to verify the targeting effect of miR-503 on RAF. The results showed that miR-503 was downregulated in a dose- and time-dependent manner in PDGF-BB-induced FBs. In addition, RAF is a direct target of miR-503 and can be negatively regulated. Overexpression of RAF can promote FB proliferation, migration, differentiation, collagen synthesis, and activation of downstream molecules (MEK/ERK), while overexpression of miR-503 can partially reverse the effects of RAF. Therefore, miR-503 regulates the biological behavior of PDGF-BB-induced oral mucosal FBs by influencing the activation of the RAS/RAF/MEK/ERK signaling pathway.

## 1. Introduction

Oral submucous fibrosis (OSF) is a chronic, progressive, and carcinomatous scar disease of the oral mucosa [1]. Depending on the cause of the disease, Sharma, M. et al. [2] subdivided OSF into areca nut-induced oral fibrosis (AIOF) to distinguish it from other forms of fibrosis in the oral region. The malignant conversion rate of OSF is as high as 1.5–15%, which greatly increases the mortality rate, and patients often have clinical symptoms such as dry mouth, ulcers, progressive mouth restriction, and dysphagia, which significantly reduces the survival rate and quality of life of patients [3,4]. Therefore, it is particularly urgent to find effective prevention and treatment methods for OSF.

The pathological features of OSF are abnormal proliferation and differentiation of oral mucosal fibroblasts (FBs) into myofibroblasts (MFBs). This eventually leads to excessive deposition of extracellular matrix protein (ECM) such as collagen [5]. Oral mucosa FB is the main effector cell of OSF, and its continuous activation is dependent on the changes in the immune microenvironment, such as cytokines. Platelet-derived growth factor (PDGF) is a potent fibroblast stimulator with five biologically active proteins, including PDGF-AA, PDGF-BB, PDGF-CC, PDGF-DD, and PDGF-AB [6]. Among them, PDGF-BB plays an important role in fibrotic diseases. Our previous studies [7,8] found that PDGF-BB could induce the proliferation of oral mucosal FBs and their differentiation into MFBs, and MFBs showed enhanced proliferation, migration, and collagen generation capabilities, thus promoting oral submucosal tissue fibrosis. However, the exact pathogenesis of PDGF-BB regulating oral mucosal FBs has not been well elucidated. Therefore, looking for new molecular mechanisms for PDGF-BB-induced oral mucosal FBs may be a feasible strategy for developing new anti-OSF drugs.

MicroRNA (miRNA/miR) is a class of endogenous non-coding RNA with a length of about 18–22 nucleotides. It can negatively regulate the expression of target genes after transcription by binding to the 3′untranslated region (3′UTR) of target genes and participate in a variety of physiological processes [9,10]. In recent years, more and more reports have shown that abnormal expression of miRNA is closely related to tumors, cardiovascular diseases, and a variety of fibrotic diseases [11]. Studies have confirmed that multiple miRNA molecules, including miR-203, miR-200c, miR-21, and miR-10b, are involved in the regulation of FBs proliferation and activation, collagen synthesis, and epithelial–mesenchymal cell differentiation in OSF tissues, indicating that miRNA plays an important role in the occurrence and development of OSF [12,13,14,15]. Therefore, we posed a scientific question: Is there a relationship between PDGF-BB and miRNA in oral mucosa FBs in the progression of OSF?

Bi et al. [16] found that PDGF-BB can downregulate the expression of miR-503 in vascular smooth muscle cells in atherosclerotic diseases, and evidence suggests that miR-503 can be used as a tumor suppressor. Database retrieval also found that its expression in oral cancer tissues was significantly reduced, indicating that miR-503 may be an important regulator of the occurrence and development of oral cancer [17,18]. In addition, miR-503 is also an important fibrotic regulator in various fibrotic diseases. In liver fibrosis, Xie et al. [19] found that miR-503 was involved in the regulation of the TGF-β/SMAD pathway by targeting SMAD7 to promote hepatic stellate cell activation and liver fibrosis. Similarly, abnormal regulation of miR-503 can lead to various pathological changes such as angiogenesis, myocardial fibrosis, and oxidative stress, which in turn aggravates the severity of cardiovascular disease [20]. Wu et al. [21] found that miR-503 synergistically targets VEGFA and FGFR1 blocks MAPK/ERK signaling, delays FB activation and MFB differentiation, and thus attenuates silica-induced pulmonary fibrosis. Christoph et al. [22] found in the model of renal fibrosis in vitro that miR-503 can cause the inactivation of calcium-dependent potassium channel protein (KCNN4) channel dependent on renal fibroblasts and inhibit cell proliferation, thus playing a role in inhibiting renal fibrosis. At the same time, it has been reported that the binding of PDGF-BB to the receptor can cause the activation of Ras, proto-oncogene serine/threonine protein kinase (RAF1), bispecific mitogen-activated protein kinase (MEK) and extracellular signal-regulated protein kinase (ERK) cascade signaling pathways, therefore promoting the proliferation, activation and differentiation of FBs and playing a role in promoting fibrosis [23,24,25].

In conclusion, the main purpose of this study was to investigate the regulatory effects of miR-503 on the proliferation, migration, and differentiation of oral mucosal FBs induced by PDGF-BB and its molecular mechanism. This will help to clarify the role of PDGF-BB in the pathogenesis of OSF and the possibility of miR-503 as a new therapeutic drug. It provides a new theoretical and experimental basis for revealing the pathogenesis of OSF and improving the therapeutic effect of OSF.

## 2. Materials and Methods

### 2.1. Cell Culture, Grouping and Processing

The experimental method of isolation and extraction of human primary oral mucosa FBs was carried out according to the previous operating process [7,8]. The cells were cultured in cell culture medium (DMEM) containing 15% fetal bovine serum (FBS, Gibco, Grand Island, NY, USA), 100 U/mL penicillin, and 100 μg/mL streptomycin (Beyotime Bio, Shanghai, China). Culture condition: 37 °C 5% CO_2_.

FBs at the logarithmic growth stage were randomly divided into 3 groups: blank group, experimental group, and transfection group. The blank group consisted of normal cultured oral mucosa FBs. The experimental group was treated with PDGF-BB (PEPROTECH, East Windsor, NJ, USA) for 24 h. The transfection group was configured with 100nM miR-503 NC and miR-503 mimic transfection mixture according to the instructions of the riboFECT™CP kit (RiboBio Co., Guangzhou, China). The transfection reagent mixture of 100pmol miR-503 mimic and 2.5 μg pcDNA-RAF was prepared according to the instructions of the lipo8000 transfection kit. After incubation at room temperature for 15–20min, the transfection reagent mixture was uniformly added to 2ml fresh medium for 16 h, then the old medium was discarded, and the cells were treated with conditioned medium containing PDGF-BB (DMEM medium containing 10% fetal bovine serum) for 24 h. The construction of miR-503 NC and miR-503 mimic was completed by Ruibo Biotechnology Co., LTD. The plasmid that overexpressed RAF (pcDNA3.1-3xFlag-C/RAF1) was purchased from Shanghai Jikai Gene Technology Co., LTD (Shanghai, China).

### 2.2. Real-Time Fluorescence Quantitative PCR (RT-qPCR) Experiment

According to the manufacturer’s instructions, TRIzol reagent (Yeasen, Shanghai, China) was used to lyse and extract total RNA from oral mucosal fibroblasts. Then, according to the standard of the cDNA reverse transcription kit (Yeasen, Shanghai, China), 500 ng total RNA (10 uL) was added into the reverse transcription system to obtain cDNA. The mRNA levels were detected by Hieff qPCR SYBR Green Master Mix (Yeasen, Shanghai, China) and RT-qPCR (Applied Biosystems 7500, Foster City, CA, USA). The reaction conditions of qPCR were predenaturation at 95 °C for 5 min, denaturation at 95 °C for 10 s, annealing/stretching at 60 °C for 34 s, and a total of 40 cycles. Three repeat holes were set for each sample. U6 or glyceraldehyde 3-phosphate dehydrogenase (GAPDH) was used as an internal control, and the relative expression of mRNA was calculated by the 2^−ΔΔCt^ method. The primer sequence is shown in Table 1.

### 2.3. Western Blot Test

Total intracellular protein was extracted from RIPA cell lysate containing a protease inhibitor, and the concentration of protein samples was quantitatively detected by the BCA protein assay kit (Bioss, Beijing, China). The 40 μg boiled denatured protein sample was separated on 10% sodium dodecyl sulfate–polyacrylamide gel electrophoresis (SDS–PAGE) and transferred to a polyvinylidene fluoride (PVDF) membrane. The membrane was soaked in 5% skim milk closed for 1 h and incubated with a specific primary antibody at 4 °C overnight. Then, the membrane was washed three times with PBS containing 0.1% Tween 20 and incubated with enzyme-labeled secondary antibody at 37 °C for 1 h in the dark, and the membrane was washed again. Finally, the enhanced chemiluminescent substrate (ECL) system showed protein bands, and the gray values of each band were determined by Image J 1.53K software (National Institute of Health, Bethesda, MD, USA) for quantitative analysis. The main antibody dilution ratios were as follows: primary antibody (GAPDH 1:3000, COL-I 1:1000, α-SMA 1:500, RAF 1:500, p-ERK 1:300, p-MEK 1:500), secondary antibody (HRP-Sheep against rabbit 1:5000, HRP-sheep against mouse 1:5000).

### 2.4. Cell Counting Kit-8 (CCK-8)

Cell counting kit 8 (CCK-8) was used to detect the viability of oral mucosal fibroblasts, results of this assay are often used to determine cell proliferation indirectly. The transiently transfected oral mucosal fibroblast suspension (1 × 10^4^/well) was seeded in 96-well plates and cultured overnight at 37 °C in a 5% CO_2_ cell incubator. After the cells were adherent, the old medium was discarded. The cells were cultured in the conditioned medium containing 80 ng/mL PDGF-BB for 0, 12, 24, 48, 72, 96 h, respectively, and 5 mg/mL CCK-8 reagent 10 uL/well was added. The cells were cultured in the incubator at 37 °C for 2 h. The absorbance at 450nm of each pore was determined by an enzyme-labeled method. Cell viability (%) = [OD (dosing) − OD (blank)/OD (0 dosing) − OD (blank)] × 100%. Absorbance is proportional to cell viability, and the final data are expressed as a percentage relative to control cells.

### 2.5. Cell Scratch Assay

Cell migration ability was evaluated using cell scratch assay. The cells were uniformly inoculated into the six-well plate and cultured with conditioned media until the cell density reached 80–90%. The cell culture medium in each group was discarded on the cell operating table, and 3 scratches were made with a sterile 200 uL TIP tip perpendicular to the bottom of the culture plate at a uniform speed. PBS is added quickly after each hole is completed, and the time interval is not more than 1 min to avoid cell death due to drying. The exfoliated cells were gently washed with PBS, and serum-free medium was added to each group of cells to continue the culture. The migration of cells in each group was observed under an inverted microscope at 0, 6, 12, 24, 36, 48, and 72 h. The scratch area of each group was measured by ImageJ software. The average relative migration rate of cells at each measurement point was calculated according to the formula migration rate (%) = [(initial scratch area − scratch area at the corresponding time point)/initial scratch area] × 100%.

### 2.6. Double Luciferase Reporter Gene Experiment

Wild-type (WT-) or mutant (MUT-) RAF 3′UTR was synthesized, expanded, and inserted downstream of the firefly luciferase reporter gene in the psiCHECK-2 vector by Rube Biotechnology. The cells were uniformly inoculated in the 24-well plate, 100 nm pmiRGLO-RAF-WT or pmiRGLO-RAF-MUT and 100 nm miR-503 mimic or miR-503 mimic control (miR-503 NC) were co-transfected with lipofectamine 2000 transfection reagent, and cells were collected 48 h later. According to the instructions of the double luciferase reporter test kit (Transgen, Beijing, China), the activity of firefly luciferase (firefly) and Renilla luciferase (Renilla) were measured on the chemiluminescence instrument, and each hole was normalized.

### 2.7. The Cancer Genome Atlas Program (TCGA)

TCGA collects clinical data, genomic variation, mRNA expression, methylation, and other data on various human cancers and is one of the data sources for cancer research. It can search the database and obtain information through the official website (https://portal.gdc.cancer.gov). TCGA (accessed on 15 May 2022) was used to compare the expression of miR-503 in oral squamous cell carcinoma tissues and normal tissues to explore whether there were differences. Expression and the survival curve of miR-503 in head and neck squamous cell carcinoma tissues were then generated online.

### 2.8. Other Databases

TargetScan [26] (https://www.targetscan.org/vert_80/), miRanda [27,28] (http://mirtoolsgallery.tech/mirtoolsgallery/node/1055), and starBase [29] (https://rnasysu.com/encori/) (accessed on 22 October 2022) database were used to search for potential target genes of miR-503 to inhibit the abnormal proliferation, migration, and differentiation of FBs in oral mucosa induced by PDGF-BB.

### 2.9. Statistical Analysis

In this study, the GraphPad Prism version 9.0.0 software (GraphPad Software, Boston, MA, USA, www.graphpad.com) was used for statistical analysis of the data of each group and the result graph was drawn. All results were expressed as mean ± standard deviation (SD), and differences between groups were measured by paired sample *t*-test with a confidence interval of 95%. *p* < 0.05 indicated statistically significant differences.

## 3. Results

### 3.1. PDGF-BB Decreased the Expression of miR-503 in Oral Mucosal Fibroblasts

**Figure 1:**
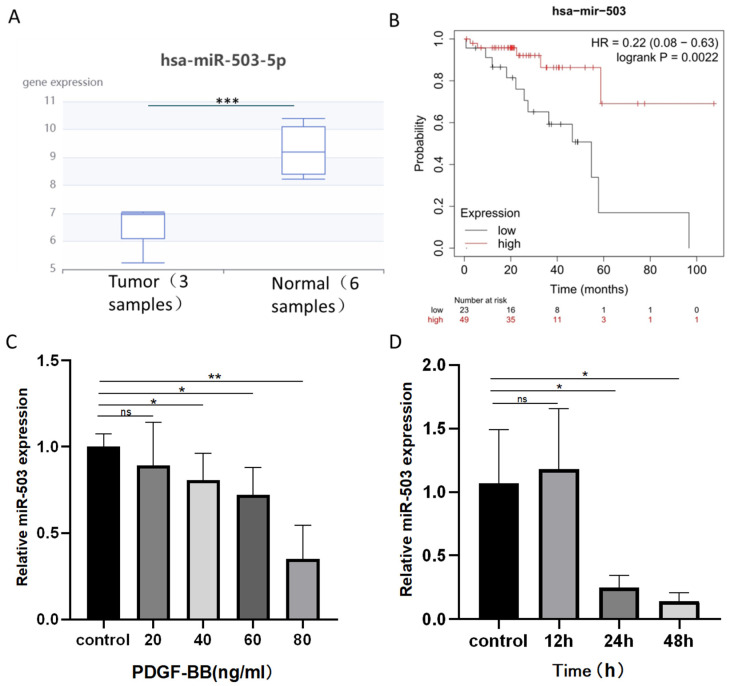
PDGF-BB decreased the expression of miR-503 in oral mucosal fibroblasts. (**A**): A search of the database showed that miR-503 was less expressed in oral squamous cell carcinoma tissues compared with normal controls. (**B**): The Kaplan–Meier curve was used to analyze the relationship between miR-503 expression and survival in head and neck squamous cell carcinoma tissues in the TCGA database. (**C**): RT-qPCR was used to detect the expression level of miR-503 in oral mucosal FBs stimulated by different concentrations of PDGF-BB (0, 20, 40, 60, 80 ng/mL) for 24 h, and the mRNA expression level of U6 was used as the internal control. (**D**): After 0, 12, 24, and 48 h stimulation by PDGF-BB (80 ng/mL), the expression level of miR-503 and the mRNA expression level of U6 were detected by RT-qPCR. (Compared with the control group, * meant *p* < 0.05, ** meant *p* < 0.01, *** *p* < 0.001, ns means not significant).

A search of the TCGA database showed that the expression of miR-503 in oral squamous cell carcinoma tissues was significantly downregulated compared with para-cancerous normal tissues (Figure 1A, *p* < 0.001). Further search revealed that in cases of head and neck squamous cell carcinoma, the overall survival rate of patients in the group with high expression of miR-503 was higher than that in the group with low expression of miR-503 (Figure 1B, *p* < 0.01), suggesting that miR-503 may be an important regulator of the development of oral squamous cell carcinoma. Our previous studies have shown that PDGF-BB can induce key pathological processes such as abnormal activation and differentiation of oral mucosa FBs and is an effective regulator of OSF. To detect whether the expression of miR-503 in oral mucosa FBs induced by PDGF-BB is regulated, we stimulated oral mucosal FBs with different concentrations of PDGF-BB (0, 20, 40, 60, 80 ng/mL) and then detected the expression of miR-503 by RT-qPCR. The results showed that with the increase of PDGF-BB stimulation concentration, the expression of miR-503 in FBs was significantly decreased, showing a significant dose-dependent trend, and the expression level of miR-503 after 80 ng/mL PDGF-BB stimulation was significantly lower than that in 0, 20, 40 and 60 ng/mL groups. The difference was statistically significant (Figure 1C). In addition, RT-qPCR was used to detect the expression of miR-503 in oral mucosal FBs stimulated by PDGF-BB (80 ng/mL) for 0, 12, 24, and 48 h. The results showed that the expression of miR-503 in oral mucosal FBs stimulated by PDGF-BB decreased significantly with the extension of stimulation time, and the expression of miR-503 in FBs was significantly lower than that at 0 h and 12 h after 24 h and 48 h stimulation by PDGF-BB (80 ng/mL), with statistically significant differences (Figure 1D). These results indicated that PDGF-BB could reduce the expression of miR-503 in oral mucosa FBs with a certain time-dose-dependent effect. Moreover, 80 ng/mL PDGF-BB was selected to treat oral mucosal FBs for 24 h for the follow-up experiment.

### 3.2. Overexpression of miR-503 Attenuates the Induction Effect of PDGF-BB on Oral Mucosal Fibroblast Differentiation

**Figure 2:**
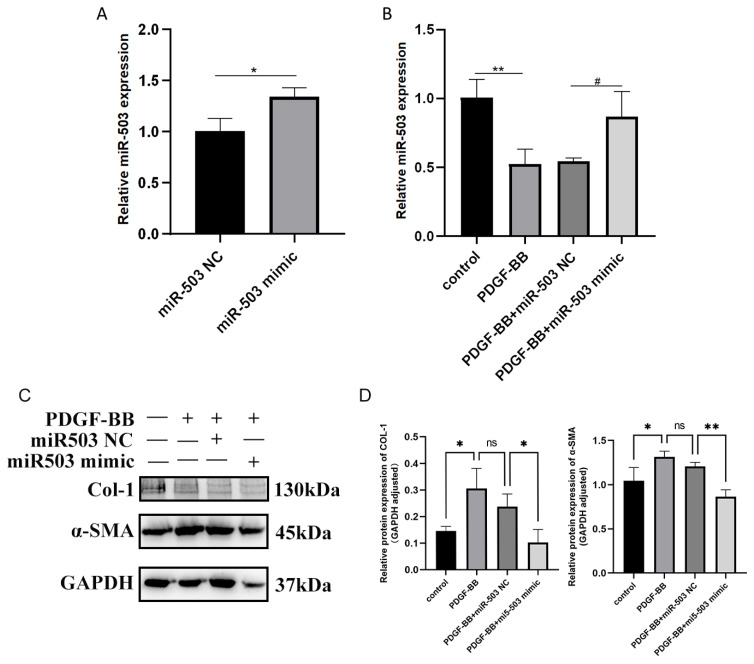

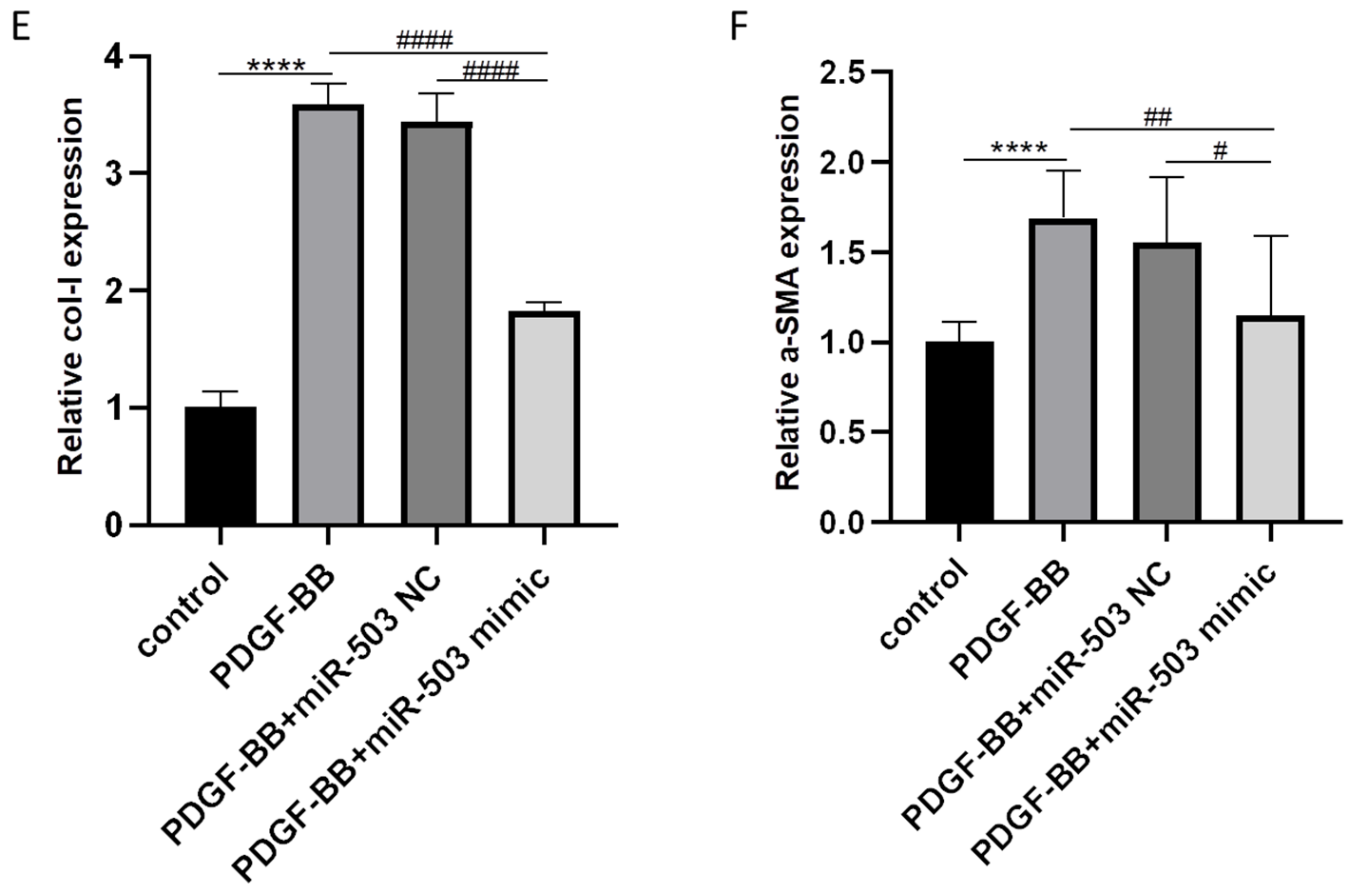
Overexpression of miR-503 inhibits the expression of Col-I and α-SMA in PDGF-BB-induced oral mucosal fibroblasts. (**A**): The expression level of miR-503 in oral mucosal FBs transfected with miR-503 mimic was detected by RT-qPCR. NC: negative control; (**B**): The expression level of miR-503 in oral mucosal fibroblasts transfected with miR-503 mimic stimulated by PDGF-BB was detected by RT-qPCR. The mRNA expression level of U6 was used as the internal control. (**C**): Western blot analysis of the effects of overexpression of miR-503 on the expression levels of Col-I and α-SMA proteins in PDGF-BB-induced oral mucosal FBs. (**D**): Statistical results of intracellular Col-I and α-SMA protein expression levels under the action of overexpression of miR-503 and PDGF-BB. (**E**): The effect of overexpression of miR-503 on Col-Ⅰ mRNA expression level in PDGF-BB-induced oral mucosal FBs was detected by RT-qPCR, and the mRNA expression level of U6 was used as the internal control. (**F**): The effect of overexpression of miR-503 on α-SMA mRNA expression level in PDGF-BB-induced oral mucosal FBs was detected by RT-qPCR, and the mRNA expression level of U6 was used as the internal control. (Compared with the control group, * means *p* < 0.05, ** means *p* < 0.01, **** means *p* < 0.0001; Compared with PDGF-BB + miR-503 mimic group, # indicated *p* < 0.05, ## indicated *p* < 0.01, and #### indicated *p* < 0.0001). Original Western blot images can be found in Supplementary File S1.

Activation and differentiation of FBs into MFBs is an important process in the development of OSF. In the process of cell differentiation, MFBs have a significantly stronger ability to synthesize collagen (Col-I) than fibroblasts, and α-SMA is only expressed in MFBs. To investigate the regulatory role of miR-503 after PDGF-BB-induced FBs differentiation in oral mucosa, miR-503 mimic and its negative control (miR-503 NC) were transfected into oral mucosal FBs, and the expression level of miR-503 was detected by RT-qPCR. The results showed that the expression of miR-503 in oral mucosa FBs was significantly upregulated and successfully overexpressed in oral mucosa FBs (Figure 2A). RT-qPCR was used to detect the expression of FBs after transfecting miR-503 mimic/NC into oral mucosa stimulated by PDGF-BB. The results showed that compared with the control group, PDGF-BB stimulation could reduce the expression of miR-503 in oral mucosa FBs. In the oral mucosa FBs stimulated by PDGF-BB, the expression level of miR-503 gene in the group transfected with miR-503 mimic was higher than that in the group transfected with miR-503 NC, and the differences were statistically significant (Figure 2B). These results indicated that miR-503 was successfully overexpressed in oral mucosa FBs, and PDGF-BB stimulation could partially reduce the overexpression of miR-503 in oral mucosa FBs.

The effect of overexpression of miR-503 on the expression of fibrosis-related proteins Col-I and α-SMA in oral mucosa FBs induced by PDGF-BB was detected by Western blot. The results showed that compared with the control group, Col-I and α-SMA protein levels were upregulated in PDGF-BB stimulated oral mucosa FBs, and the expressions of Col-I and α-SMA in cells of PDGF-BB + miR-503 mimic group were significantly lower than those of PDGF-BB + miR-503 NC group (Figure 2C,D). The gene expression levels of Col-I and α-SMA in cells of each group were further detected by RT-qPCR. The results were consistent with the above results. Overexpression of miR-503 decreased the induction effect of PDGF-BB on Col-I and α-SMA expression in oral mucosa FBs. The expression of Col-I and α-SMA mRNA was significantly inhibited (Figure 2E,F). These results suggest that PDGF-BB can increase the synthesis of intracellular fibrosis-related proteins Col-I and α-SMA, induce the activation and differentiation of oral mucosal FBs into MFBs, and promote fibrosis. Overexpression of miR-503 can inhibit the induction of Col-I and α-SMA in oral mucosa FBs by PDGF-BB, indicating that miR-503 can weaken the induction of PDGF-BB on the activation and differentiation of oral mucosa FBs and has antifibrotic properties.

### 3.3. Overexpression of miR-503 Can Reduce the Proliferation and Migration of Oral Mucosa Fibroblasts Induced by PDGF-BB

**Figure 3:**
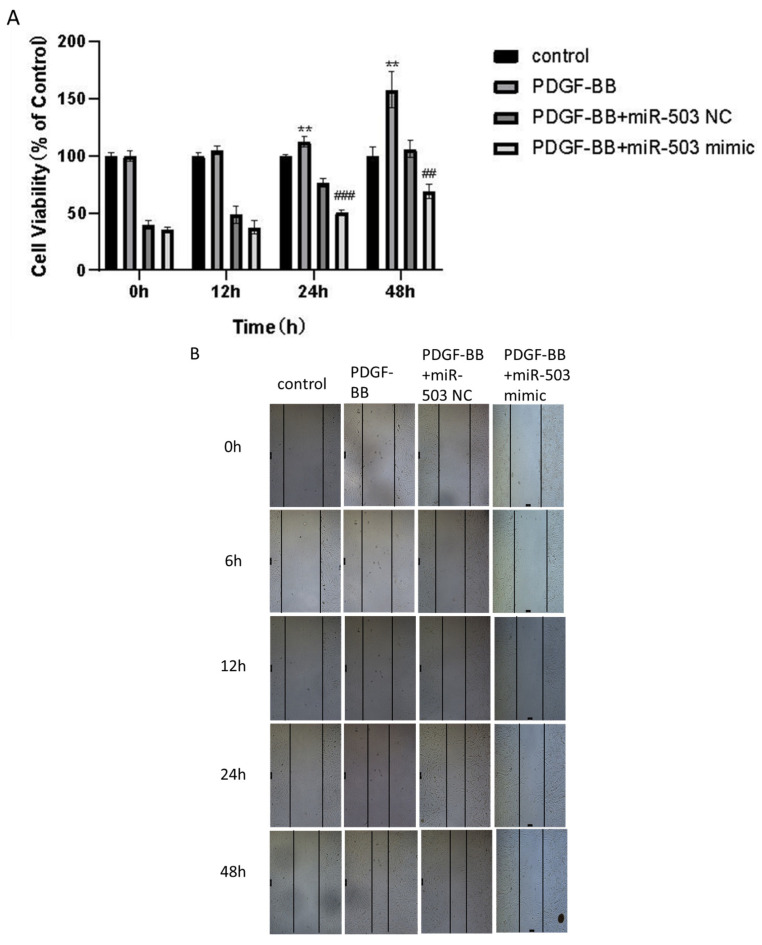

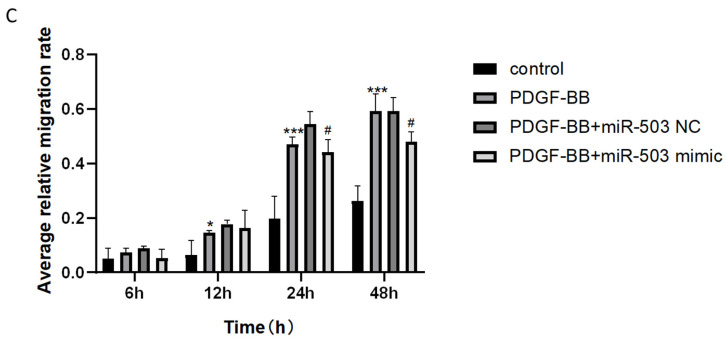
Overexpression of miR-503 inhibits the proliferation and migration of PDGF-BB-induced oral mucosal fibroblasts. (**A**): The proliferation capacity of oral mucosal fibroblasts was detected by CCK-8 assay. After transfecting miR-503 mimic or miR-503 NC into oral mucosal FBs, the cells were treated with 80 ng/mL PDGF-BB and cultured for 0, 12, 24, and 48 h. Cell OD values of the control group, PDGF-BB group, PDGF-BB + miR-503 mimic group, PDGF-BB + miR-503 mimic group, and the regulation group were measured, respectively. Cell viability (%) calculating formula for [OD (dosing) − OD (blank)/OD (0 dosing) − OD (blank)] × 100%. (**B**): The migration ability of oral mucosal fibroblasts was detected by scratch test. The cells were scratched and photographed, and the relative migration width of the cells in the control group, PDGF-BB group, PDGF-BB + miR-503 NC group, and PDGF-BB + miR-503 mimic group were measured (×50). (**C**): The average relative mobility of each group after 6, 12, 24, and 48 h of cell culture; The average cell migration rate (%) was calculated as [(cell gap at 0 h − cell gap after 6/12/24/48 h)/cell gap at 0 h] × 100%. (Compared with the control group, * means *p* < 0.05, ** means *p* < 0.01, *** means *p* < 0.001; Compared with PDGF-BB + miR-503 mimic group, # indicated *p* < 0.05, ## indicated *p* < 0.01, ### indicated *p* < 0.001).

Activation effects such as abnormal proliferation and migration of oral mucosal FBs are important phenotypes of oral submucosal fibrosis. CCK-8 is an indirect test of cell proliferation by measuring cell viability (number of living cells). Therefore, to further investigate whether miR-503 upregulation plays a role in the proliferation of oral mucosal FBs induced by PDGF-BB, we used CCK-8 assay to detect the effect of transfection of miR-503 mimic on the viability of FBs induced by PDGF-BB in the oral mucosa. The oral mucosa FBs in the logarithmic growth stage were inoculated into 96-well plates with a density of 1 × 10^4^ cells/well and cultured with conditioned medium containing PDGF-BB (80 ng/mL) for 0, 12, 24, and 48 h, respectively, to detect the absorbance (OD_450_) value of oral mucosa FBs in each group, and reflect the cell proliferation by cell vitality. The cell proliferation of control, PDGF-BB, PDGF-BB + miR-503 NC, and PDGF-BB + miR-503 mimic groups was compared. The results showed that compared with the control group, the proliferation capacity of FBs in oral mucosa was significantly upregulated after PDGF-BB stimulation for 24 and 48 h, and the difference was statistically significant. The proliferation ability of FBs in the oral mucosa of the PDGF-BB + miR-503 mimic group was significantly lower than that of the PDGF-BB + miR-503 NC group, and the difference was statistically significant (Figure 3A). The results indicated that overexpression of miR-503 could inhibit the proliferation of FBs in oral mucosa induced by PDGF-BB.

In addition, the effect of transfection of miR-503 mimic on PDGF-BB induced FBs migration in oral mucosa was examined by cell scratch assay. The average scratch width was calculated to evaluate the migration ability of the cells. The results showed that with the increase of culture time, the scratch width of all groups decreased gradually. After 12, 24, and 48 h of cell culture, the scratch width of the PDGF-BB group was significantly smaller than that of the control group, while the scratch width of the PDGF-BB + miR-503 mimic group was significantly wider than that of the PDGF-BB and PDGF-BB + miR-503 NC group (Figure 3B). After analyzing the mean relative migration rate of cells by software data, it was found that the mean relative migration rate of cells in the PDGF-BB group was significantly higher than that in the control group after 12, 24, and 48 h of cell culture. The mean relative mobility of cells of the PDGF-BB + miR-503 mimic group was lower than that of the PDGF-BB + miR-503 NC group, and the difference was statistically significant (Figure 3C), indicating that overexpression of miR-503 inhibited the induction of FBs migration ability of PDGF-BB in the oral mucosa. These results indicated that PDGF-BB could induce the proliferation and migration of FBs in oral mucosa and promote fibrosis, while miR-503 could weaken the promoting effect of PDGF-BB on cell proliferation and migration.

### 3.4. RAF Is the Direct Target of miR-503 and Can Be Negatively Regulated by IT

**Figure 4:**
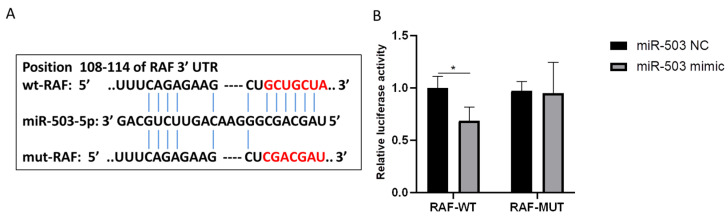

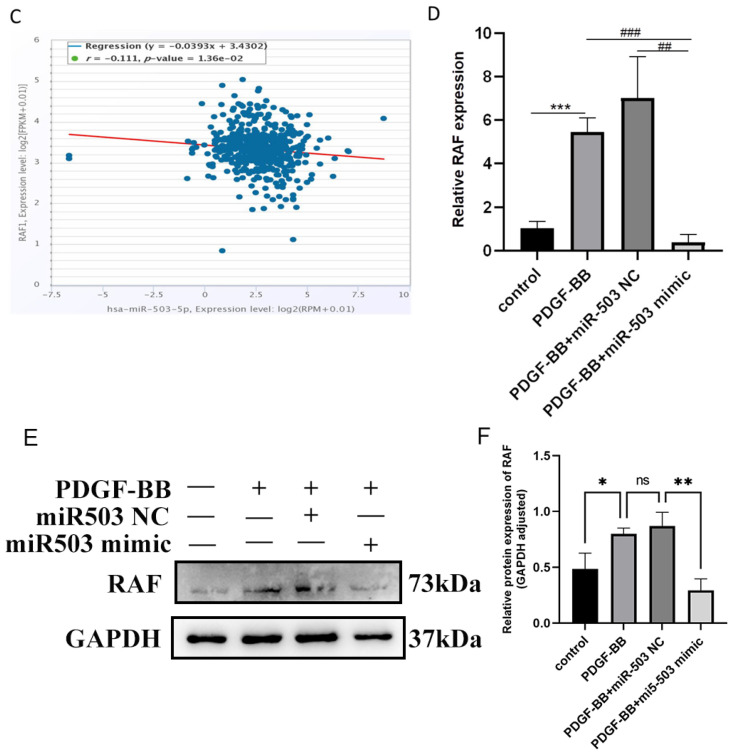
RAF is the direct target of miR-503. (**A**): Schematic diagram of binding sites of miR-503 to wild-type or mutant RAF 3′UTR. (**B**): Wild-type and mutant RAF 3′UTR luciferase reporter plasmids, namely WT-RAF and MUT-RAF, were constructed, respectively. The predicted miR-503 binding site in mut-RAF was mutated. The luciferase reporter plasmid was transfected into oral mucosal FBs with miR-503mimic or negative control, and the relative luciferase activity was measured after culture for 48 h. (**C**): The miRanda database was used to search the relationship between miR-503 and RAF gene expression in head and neck squamous cell carcinoma. (**D**): The effect of transfection of miR-503 mimic on the expression of RAF mRNA in PDGF-BB stimulated oral mucosal FBs was quantitatively detected by RT-qPCR, and the mRNA expression level of U6 was used as the internal control. (**E**): Western blot analysis of the effect of transfection of miR-503 mimic on the expression of RAF protein in PDGF-BB stimulated oral mucosal FBs. (**F**): Statistical results of the expression level of RAF protein in cells under overexpression of miR-503 and PDGF-BB. (Compared with the control group, * *p* < 0.05, ** *p* < 0.01, *** means *p* < 0.001; Compared with the PDGF-BB + miR-503 mimic group, ## indicated *p* < 0.01, and ### indicated *p* < 0.001). Original Western blot images can be found in Supplementary File S1.

TargetScan, miRanda database, and starBase network prediction software were used to find potential target genes of miR-503 to inhibit the abnormal proliferation, migration, and differentiation of FBs in oral mucosa induced by PDGF-BB. The results showed that there was a potential binding site between miR-503 and RAF 3′UTR region (Figure 4A). To verify that RAF is a direct target of miR-503, we inserted wild-type and mutant RAF 3′UTR into a dual luciferase reporter vector, WT-RAF and MUT-RAF, respectively. The constructed recombinant plasmid was transfected into oral mucosal FBs with miR-503 mimic or negative control, and the luciferase activity was measured. The results showed that miR-503 mimic significantly inhibited the luciferase activity of WT-RAF compared with the control group. However, the luciferase activity with RAF binding site mutation lost its inhibitory effect (Figure 4B). To further verify that RAF is a direct target of miR-503, we searched the miRanda database and found that miR-503 was negatively correlated with RAF gene expression in head and neck squamous cell carcinoma tissues (Figure 4C). Subsequently, RT-qPCR and Western blot were used to detect the effects of transfection of miR-503 mimic or negative control on the expression levels of RAF mRNA and protein in oral mucosa FBs induced by PDGF-BB. The results show that compared with the control group, the expression levels of RAF mRNA and protein in the PDGF-BB group were significantly upregulated, while the expression levels of RAF mRNA and protein in cells of the PDGF-BB + miR-503 mimic group were significantly lower than those of PDGF-BB + miR-503 NC group, and the differences were statistically significant (Figure 4D–F). Overexpression of miR-503 significantly downregulated RAF mRNA and protein expression in oral mucosa FBs induced by PDGF-BB, confirming that miR-503 could negatively regulate RAF expression. These data indicate that RAF is a direct target of miR-503 in oral mucosal FBs and can be negatively regulated by it.

### 3.5. miR-503 Negatively Regulates the Induction Effect of PDGF-BB on Oral Mucosa FBs through the RAS/RAF/MEK/ERK Signaling Pathway

**Figure 5:**
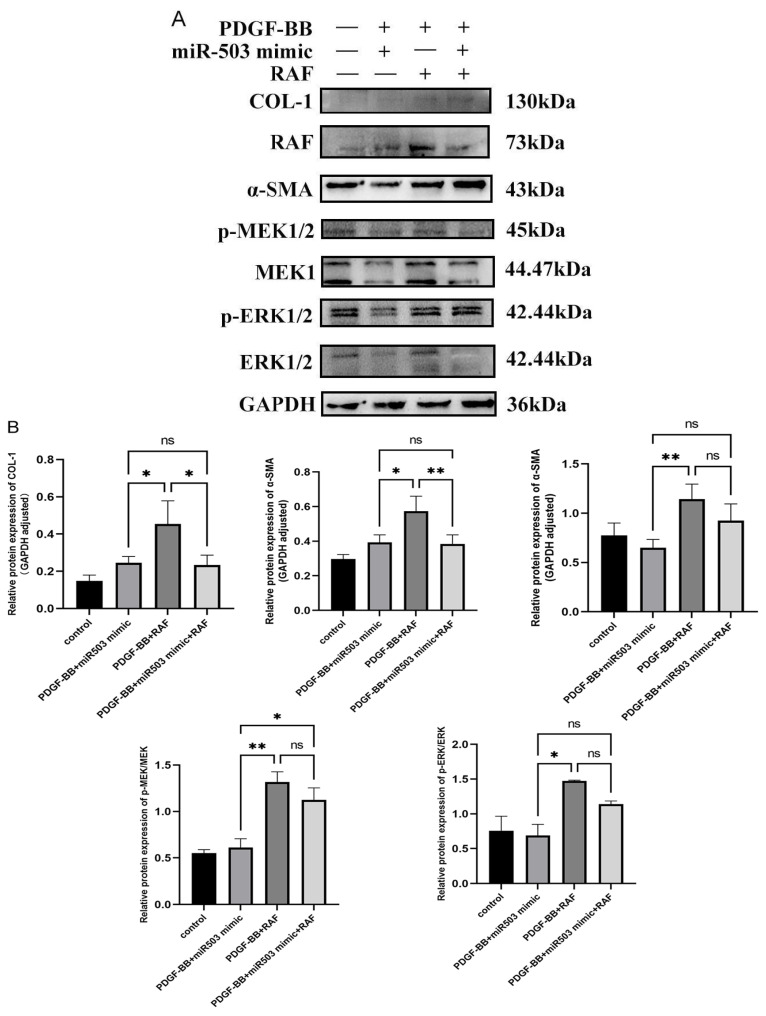

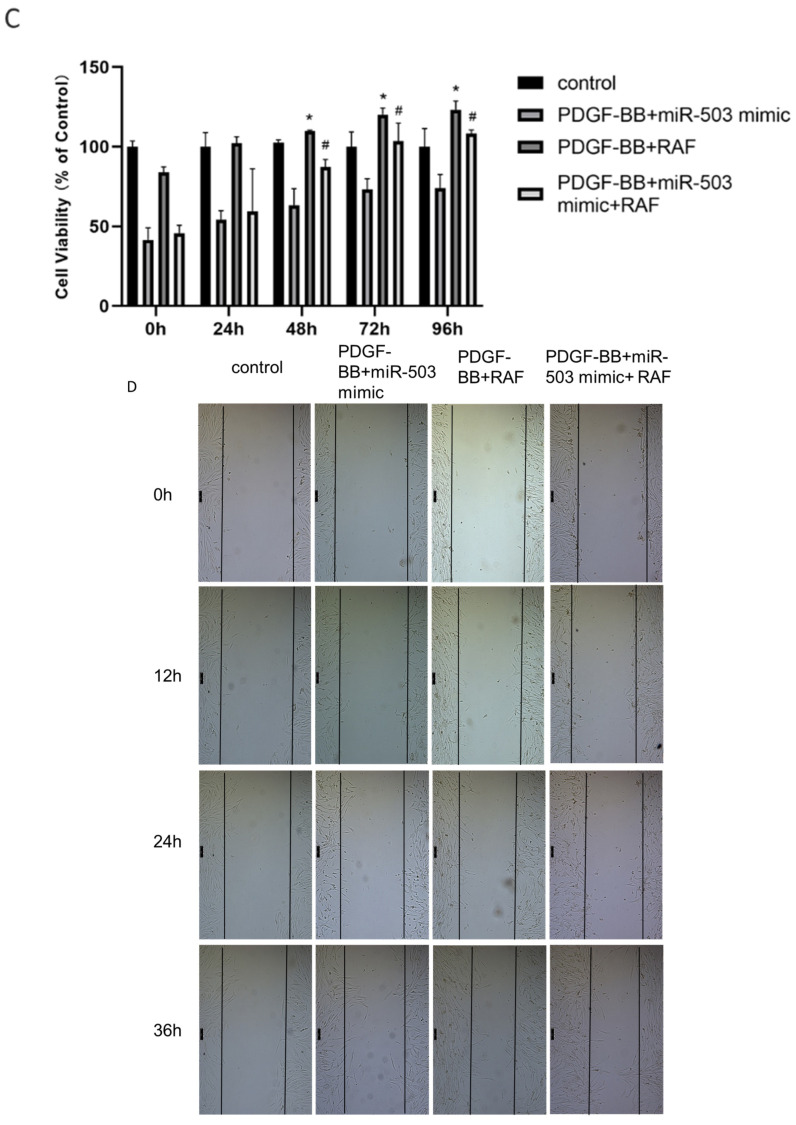

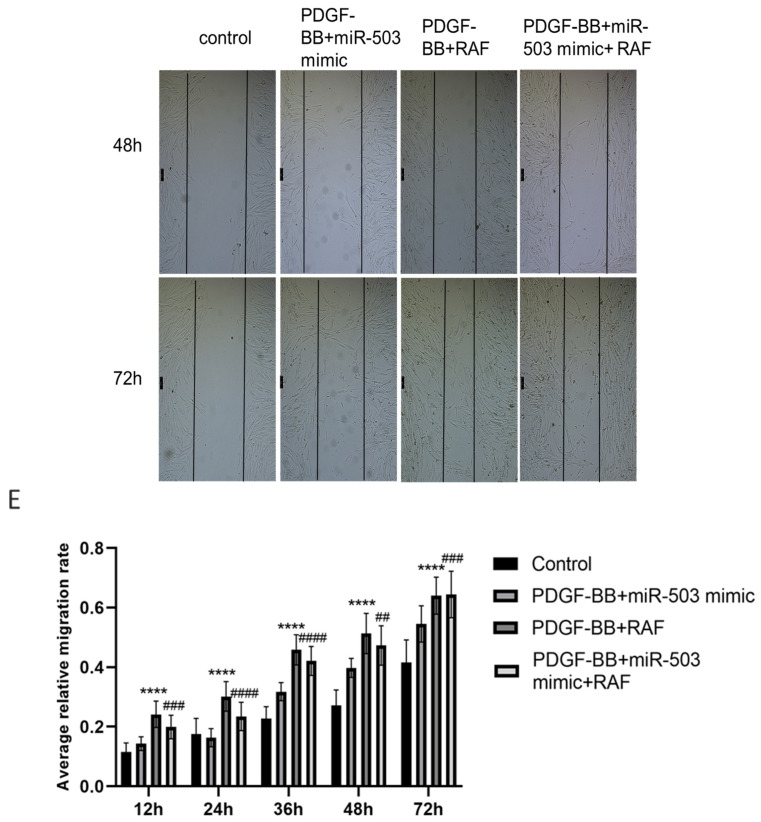
miR-503 plays an inhibitory role in regulating the RAS/RAF/MEK/ERK signaling pathway by targeting RAF. (**A**): Western blot was used to detect the effects of overexpression of miR-503 and RAF on the expression of RAS/RAF/MEK/ERK signaling pathway proteins such as RAF, p-ERK1/2, p-MEK1/2 and fibrosis markers Col-I and α-SMA in PDGF-BB-induced oral mucosal FBs. (**B**): Statistical results of expression levels of signal pathway-related proteins in PDGF-BB-induced oral mucosal FBs induced by overexpression of miR-503 and RAF. (**C**): The proliferation capacity of oral mucosal FBs was detected by CCK-8 assay. After transfecting the oral mucosal FBs with miR-503 mimic or overexpressed RAF plasmid, the cells were treated with 80 ng/mL PDGF-BB and continued to be cultured for 0, 24, 48, 72, and 96 h. Cell OD values of the control group, PDGF-BB + RAF group, PDGF-BB + miR-503 mimic group, PDGF-BB + miR-503 mimic + RAF group, and null control group were measured, respectively. Cell viability (%) calculating formula for [OD (dosing) − OD (blank)/OD (0 dosing) − OD (blank)] × 100. (**D**): The migration ability of oral mucosal FBs was detected by scratch test. The cells were scratched and photographed, and the relative migration width (×50) of the cells in the control group, PDGF-BB + RAF group, PDGF-BB + miR-503 mimic group, and PDGF-BB + miR-503 mimic + RAF group were measured. (**E**): The average relative mobility of each group after 12, 24, 48, 36, and 72 h of cell culture; The average cell migration rate (%) is calculated as [(cell gap at 0 h − cell gap after 12/24/48/36/72 h)/cell gap at 0 h] × 100%. (Compared with the control group, * means *p* < 0.05, ** means *p* < 0.01, **** means *p* < 0.0001; Compared with PDGF-BB + miR-503 mimic group, # indicated *p* < 0.05, ## indicated *p* < 0.01, ### indicated *p* < 0.001, and #### indicated *p* < 0.0001).

To further study the mechanism of miR-503 in regulating the activation of PDGF-BB-induced oral mucosal fibroblasts, i.e., whether miR-503 plays a role in inhibiting the biological behavior of FBs induced by PDGF-BB by regulating RAS/RAF/MEK/ERK signaling pathway by targeting RAF. The overexpressed RAF plasmid (pcDNA-RAF) and miR-503 mimic were transfected into oral mucosal FBs alone or together and then cultured with a conditioned medium containing PDGF-BB (80 ng/mL). Intracellular Col-I, α-SMA, and phosphorylated MEK/ERK protein expression levels, proliferation, and migration capacity were detected. Western blot results showed that Col-I, α-SMA, phosphorylated ERK (p-ERK), and p-MEK were upregulated in oral mucosa FBs after co-treatment with PDGF-BB and overexpressed RAF plasmid. However, in cells of the PDGF-BB + miR-503 mimic group, the expression of Col-I, α-SMA, p-ERK, and p-MEK was upregulated. Col-I, a-SMA, p-ERK, and p-MEK protein expressions were significantly downregulated, and the expression levels of each protein were significantly lower than those of the PDGF-BB + miR-503 mimic + RAF group (Figure 5A). The results showed that overexpression of RAF could enhance the expression of Col-I, α-SMA, RAF, p-MEK1/2, and p-ERK1/2 of PDGF-BB in oral mucosa FBs, while overexpression of miR-503 could partially reverse the effect (Figure 5B). It is suggested that overexpression of miR-503 can inhibit the expression of Col-I and α-SMA in PDGF-BB-induced oral mucosal FBs, possibly by reducing the activation of the RAS/RAF/MEK/ERK pathway.

The effect of transfection of miR-503 mimic and pcDNA-RAF on the viability of FBs induced by PDGF-BB in oral mucosa was detected by CCK-8 assay. The results showed that compared with the control group, the viability of FBs in the PDGF-BB + RAF group was significantly upregulated after 48, 72, and 96 h of cell culture. The viability of fibroblasts transfected with PDGF-BB + miR-503 mimic was significantly lower than that of those transfected with PDGF-BB + miR-503 mimic + RAF, and the difference was statistically significant (Figure 5C). The results indicated that overexpression of RAF could enhance the viability of PDGF-BB on oral mucosal FBs, while overexpression of miR-503 could reverse the effect.

In addition, the effects of transfection of miR-503 mimic and pcDNA-RAF on the migration ability of FBs induced by PDGF-BB were detected by cell scratch assay. The mean scratch width was calculated to evaluate the migration ability of cells. The results showed that with the increase of culture time, the scratch width of all groups decreased gradually. The scratch width of the PDGF-BB + RAF group was smaller than that of the control group after incubation for 12–72 h, and the scratch width of the PDGF-BB + miR-503 mimic group was larger than that of the PDGF-BB + miR-503 mimic + RAF group, and the difference increased gradually with the extension of time (Figure 5D). After 12, 24, 36, 48, and 72 h of cell culture, the mean relative migration rate of the PDGF-BB + RAF group was significantly higher than that of the control group. The mean relative mobility of cells of the PDGF-BB + miR-503 mimic group was smaller than that of the PDGF-BB + miR-503 mimic + RAF group, and the differences were statistically significant (Figure 5E). It was shown that overexpression of RAF significantly enhanced the induction effect of PDGF-BB on the migration ability of oral mucosal FBs. However, miR-503 overexpression could counteract some of the promoting effects. The above results indicated that miR-503 directly binds and negatively regulates target gene RAF, affects the activation of RAS/RAF/MEK/ERK signaling pathway, and inhibits the proliferation, migration, differentiation, and collagen synthesis of FBs in oral mucosa induced by PDGF-BB.

## 4. Discussion

The continuous activation and phenotype differentiation of oral mucosal FBs into MFBs is considered to be a key pathological process in the progression of OSF, which is manifested by high expression of smooth actin (α-SMA) and significantly enhanced collagen synthesis, cell proliferation, and migration [30,31,32]. PDGF-BB is a highly effective inducer that causes functional changes and phenotype differentiation of oral mucosa FBs, which can promote abnormal proliferation, migration, and differentiation of oral mucosa FBs into MFBs with stronger collagen synthesis ability [7,8]. More and more studies have shown that abnormally expressed miRNAs play a key role in the occurrence and development of fibrotic diseases [11], and growth factor signals such as PDGF-BB and FGF have been proven to regulate the key processes of cell proliferation and differentiation by regulating the expression of miRNA in vivo [16,33,34]. Based on the previous study of our research group, which found that inflammatory cytokine PDGF-BB can regulate the biological behavior of oral mucosa FBs [7,8], this study found that PDGF-BB can downregulate the expression of miR-503 in oral mucosal fibroblasts, and further intervention measures to overexpress miR-503 were adopted. To investigate the regulatory effects of miR-503 on the proliferation, migration, and differentiation of FBs in oral mucosa induced by PDGF-BB and its molecular mechanism. The results of this study showed that the expression of miR-503 in FBs stimulated by PDGF-BB was downregulated in a time and dose-dependent manner. Overexpression of miR-503 can inhibit the proliferation, migration, differentiation, and collagen synthesis of FBs in oral mucosa induced by PDGF-BB. In addition, we also confirmed that miR-503 could directly bind and negatively regulate the expression of RAF, therefore affecting the activation of mitogen-activated protein kinase (MAPK)/extracellular signal-regulated protein kinase 1/2 (ERK1/2) signaling pathway and inhibiting the proliferation, migration, differentiation, and collagen synthesis of FBs in oral mucosa induced by PDGF-BB.

The miR-503 is an intragenic miRNA located at the Xq26.3 locus of chromosome and belongs to the miR-15 family. It was first identified in human retinoblastoma tissues by microarray hybridization [35]. Abnormal expression of miR-503 can be seen in many types of cancer and mediates a variety of biological functions [18]. Some studies show that miR-503 has a tumor suppressor effect. Yang et al. [36] reported that miR-503 inhibited the proliferation, migration, and invasion of non-small cell lung cancer cells by downregulating the expression of target genes PI3K p85 and IKK-b. Xu et al. [37] found that miR-503 could negatively regulate cyclin D1 to inhibit the proliferation and cell cycle progression of endometrial cancer cells. Zhou et al. [38] reported that miR-503 can be used as an angiogenesis inhibitor to inhibit tumor angiogenesis and growth by simultaneously downregulating the expression of FGF2 and VEGFA. However, one study showed it to be pro-tumorigenic. In esophageal cancer, miR-503 can induce the loss of interleukin-2 and interferon-γ expression, therefore enhancing the proliferation and invasion of tumor cells [39].

Previous studies have found that miR-503 expression is downregulated in oral cancer through miRNA microarray analysis, and it can cause the cell cycle process to stop and inhibit the proliferation of head and neck squamous cell cancer cells [40,41]. This is consistent with the results of our database search, suggesting that miR-503 may be a tumor suppressor. It has been shown that miR-503 is upregulated in TGF-β1-treated oral fibroblasts [42], which may be related to antifibrotic mechanisms. In addition, a large number of studies have reported that miR-503 is abnormally expressed in various fibrotic diseases due to the regulation of cytokines and other factors. miR-503 can participate in the occurrence and development of fibrotic diseases in the heart, liver, kidney, lung, and other tissues by regulating the biological behaviors of FBs proliferation, differentiation, invasion, and migration [17,19,20]. However, the expression of FBs in oral mucosa induced by PDGF-BB remains unclear. In this study, we observed that the expression of miR-503 in oral mucosa FBs was downregulated in a time and dose-dependent manner after stimulation with PDGF-BB. Subsequently, to determine whether miR-503 was involved in regulating the biological behavior of oral mucosal FBs induced by PDGF-BB, miR-503 mimic was transfected into oral mucosal fibroblasts using intervention measures of overexpression of miR-503. Our experimental results showed that miR-503 was successfully overexpressed in oral mucosa FBs, and overexpression of miR-503 inhibited the proliferation and migration ability of oral mucosa FBs induced by PDGF-BB, and significantly reduced the expression of the signature protein of myoblasts such as Col-Ⅰ and α-SMA, suggesting that miR-503 could be used as an antifibrotic molecule. It inhibited the activation and biological behavior of FBs in oral mucosa induced by PDGF-BB. On this basis, further exploration of the target of miR-503 will help elucidate the molecular mechanism of PDGF-BB inducing fibrosis or lesions of oral mucosal FBs and provide a new possibility for targeted therapy of OSF.

Mechanistically, miRNAs perform different functions by targeting different targets. Mitogen-activated protein kinase (MAPK) is an important signal transduction system in vivo, consisting of four main branch routes: ERK, ERK5/BMK1, c-Jun N-terminal kinase (JNK), and p38MAP kinase (p38MAPK). Among them, the proto-oncogene serine/threonine protein kinase (RAF), as an important part of the classical RAS/RAF/MEK/ERK signaling pathway, is widely involved in the regulation of cell growth, development, division, differentiation, apoptosis, migration, and other cell functions [43,44]. In fibrotic diseases, the RAS/RAF/MEK/ERK signaling pathway can promote the epithelial–mesenchymal transition (EMT) of hepatic stellate cells [24]. The proliferation and activation of cardiac FBs [25], kidney FBs [22], and lung FBs [45], as well as collagen synthesis, mediate ECM deposition. In addition, previous studies have proved that PDGF-BB can activate multiple signaling pathways in various cell lines and tissues, among which RAS/RAF/MEK/ERK is the downstream signaling pathway of PDGF-BB, which is involved in regulating the abnormal growth, differentiation, apoptosis and other biological behaviors of FBs s in the course of various fibrotic diseases [46]. Our study found that the expression of RAF mRNA and protein increased in the oral mucosa FBs treated with PDGF-BB. At the same time, we searched the database and found that there was a binding site between miR-503 and the 3′UTR region of RAF, and the expression of miR-503 and RAF was negatively correlated in head and neck squamous cell carcinoma tissues. Therefore, we hypothesized that miR-503 could affect the activation of the RAS/RAF/MEK/ERK signaling pathway by targeting RAF and negatively regulating the abnormal response of FBs induced by PDGF-BB in oral mucosa, therefore alleviating the OSF.

In this study, through a double luciferase reporter gene experiment, we found that overexpression of miR-503 could significantly inhibit the luciferase activity of WT-RAF, while the luciferase activity of RAF binding site mutation was lost. It was confirmed that RAF could bind to miR-503 and was the direct target of miR-503. Meanwhile, our experimental results showed that overexpression of miR-503 could inhibit the expression of RAF mRNA and protein in oral mucosa FBs induced by PDGF-BB and negatively regulate the expression level of RAF, which was consistent with the prediction of Mann et al. [22]. These results indicate that RAF is a direct target of miR-503 in oral mucosa FBs and can be negatively regulated by it. Many studies have shown that the activation of the RAS/RAF/MEK/ERK signaling pathway induced by PDGF-BB plays an important role in the proliferation, apoptosis, migration, and differentiation of FBs [47]. Our results also found that intracellular overexpression of miR-503 inhibited the expression level of RAF protein and the phosphorylation levels of p-MEK and p-ERK in oral mucosa FBs induced by PDGF-BB. At the same time, the expression of Col-Ⅰ and α-SMA in oral mucosa FBs induced by PDGF-BB and cell proliferation and migration were inhibited. Overexpression of RAF can upregulate the expression level of RAF protein in oral mucosa FBs induced by PDGF-BB and the phosphorylation of downstream signaling pathway proteins and enhance the promotion effect of PDGF-BB on the proliferation, migration, differentiation, and collagen synthesis of oral mucosal FBs. In addition, the co-expression of RAF and miR-503 can restore the inhibitory effect of miR-503 on PDGF-BB-induced proliferation, migration, differentiation, and collagen synthesis of oral mucosal FBs. In conclusion, the upregulation of miR-503 expression can inhibit the abnormal activation and differentiation of FBs in oral mucosa induced by PDGF-BB and can also reduce the phosphorylation level of PDGF-BB-induced signaling pathway protein, while overexpression of RAF can inhibit this effect. These results suggest that overexpression of miR-503 can affect the activation of the RAS/RAF/MEK/ERK signaling pathway by downregulating the expression of target gene RAF, therefore inhibiting the fibrosis or lesions of oral mucosal FBs induced by PDGF-BB.

**Figure 6:**
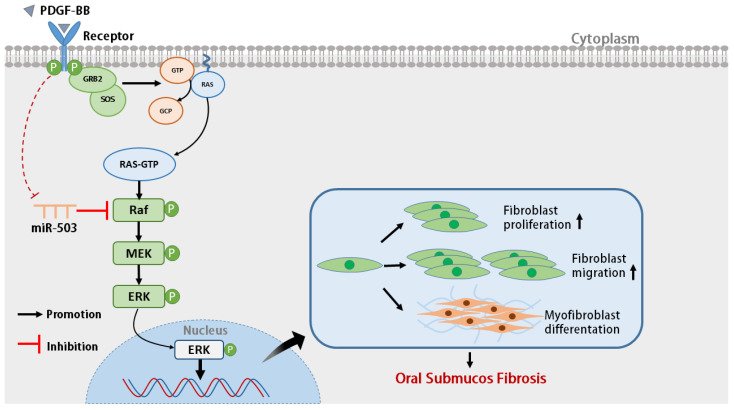
Schematic diagram of the mechanism of miR-503 regulating the biological behavior of PDGF-BB-induced oral mucosal fibroblasts. By directly binding and negatively regulating target gene RAF, miR-503 affects the activation of the RAS/RAF/MEK/ERK signaling pathway, therefore inhibiting the biological behaviors of proliferation, migration, differentiation, and collagen synthesis of oral mucosal FBs induced by PDGF-BB.

In summary, our results indicate that PDGF-BB stimulation can reduce the expression of intracellular miR-503 in oral mucosal FBs, while overexpression of miR-503 can directly bind and negatively regulate target gene RAF, affecting the activation of RAS/RAF/MEK/ERK signaling pathway. Furthermore, PDGF-BB-induced proliferation, migration, differentiation, and collagen synthesis of oral mucosal FBs were inhibited (Figure 6). Therefore, miR-503 may be a potential candidate to inhibit the activation and differentiation of FBs in oral mucosa induced by PDGF-BB, providing a new target for the antifibrotic treatment of OSF. One limitation of this study is using the cck-8 assay to determine proliferation, which cannot distinguish whether the increase in the number of viable cells was due to a stronger ability to proliferate or a decrease in apoptosis. This study still needs to conduct a more comprehensive study on the mechanism of PDGF-BB regulating the occurrence and development of OSF to provide a new experimental basis for exploring the pathogenesis and prevention of OSF, which has important scientific, social, and clinical value for the early diagnosis and treatment of OSF patients.

## 5. Conclusions

In conclusion, our research revealed that PDGF-BB enhances RAS/RAF/MEK/ERK signaling pathway activity by downregulating miR503 expression and attenuating the role of miR503 in targeting RAF, promoting the proliferation, migration, and differentiation of oral mucosa FBs.

## Figures and Tables

**Table 1:** The Primers List.

Primer	Sequence (5′ to 3′)
U6-F	CTCGCTTCGGCAGCACA
U6-R	AACGCTTCACGAATTTGCGT
hsa-miR-503-F	TGCCCTAGCAGCGGGAAC
hsa-miR-503-R	ACCCTGGCAGCGGAAACAATA
GAPDH-F	CATGGGTGTGAACCATGAGAA
GAPDH-R	GGTCATGAGTCCTTCCACGAT
Col-I-F	GTGCGATGACGTGATCTGTGA
Col-I-R	CGGTGGTTTCTTGGTCGGT
α-SMA-F	TGCCAACAACGTCATGTCG
α-SMA-R	CAGCGCGGTGATCTCTTTCT
RAF1-F	AAAGTACGACTCCCTATCCGAC
RAF1-R	GGTTGGTGGTTTCGATGGACT

## Data Availability

Data are available upon request from the corresponding authors.

## Supplementary Materials

The following supporting information can be downloaded at https://www.mdpi.com/article/10.3390/biom14101259/s1, File S1: Original Western blot images.
