# Supplementary material for: MicroRNA-503 Suppresses Oral Mucosal Fibroblast Differentiation by Regulating RAS/RAF/MEK/ERK Signaling Pathway
Source: Biomolecules. 2024 Oct 5;14(10):1259. doi: 10.3390/biom14101259 (PMC11505938; doi:10.3390/biom14101259)
Supplement: Supplementary file 1 [file biomolecules-14-01259-s001.zip › biomolecules-3022942-supplementary.pdf]

Original western blot

We cut the membrane after blocking, according to the molecular weight size from the position of 70 kDa. The membranes above 70 kDa were incubated with Col-I and RAF antibodies, and the membranes below 70 were incubated with α-SMA, MEK, p-MEK, ERK, p-ERK, and GAPDH antibodies. The membranes were labeled with the corresponding serial numbers to ensure that the portion above 70 and the portion below 70 belonged to the same membrane (Fig 1).

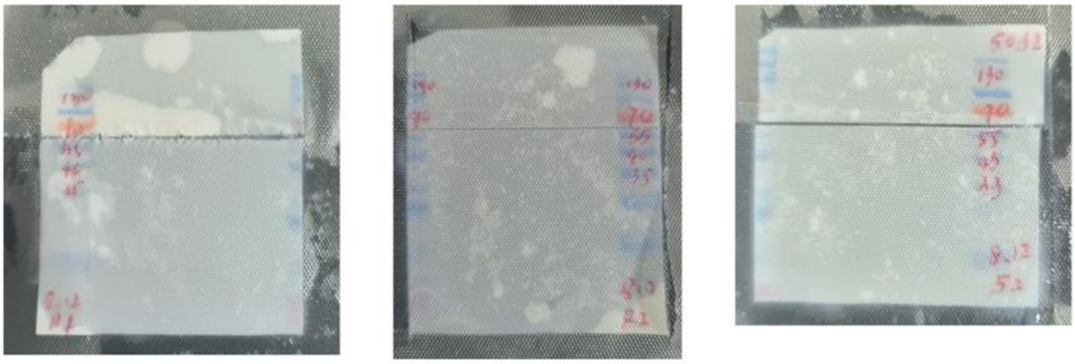

Fig1. uncropped western blots

Figure3-2 C and Figure3-4 E. Original western blot

Repeat 1

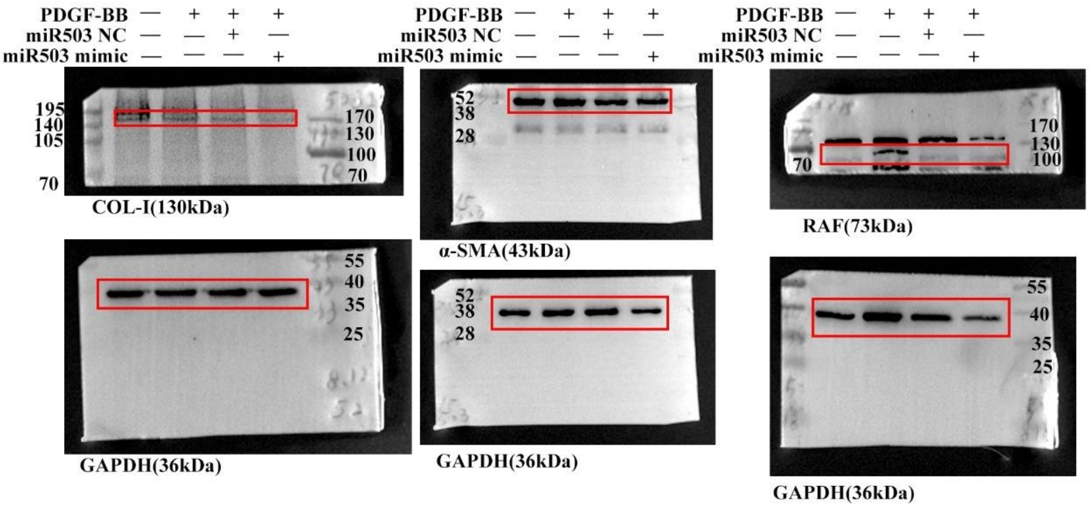

Repeat 2

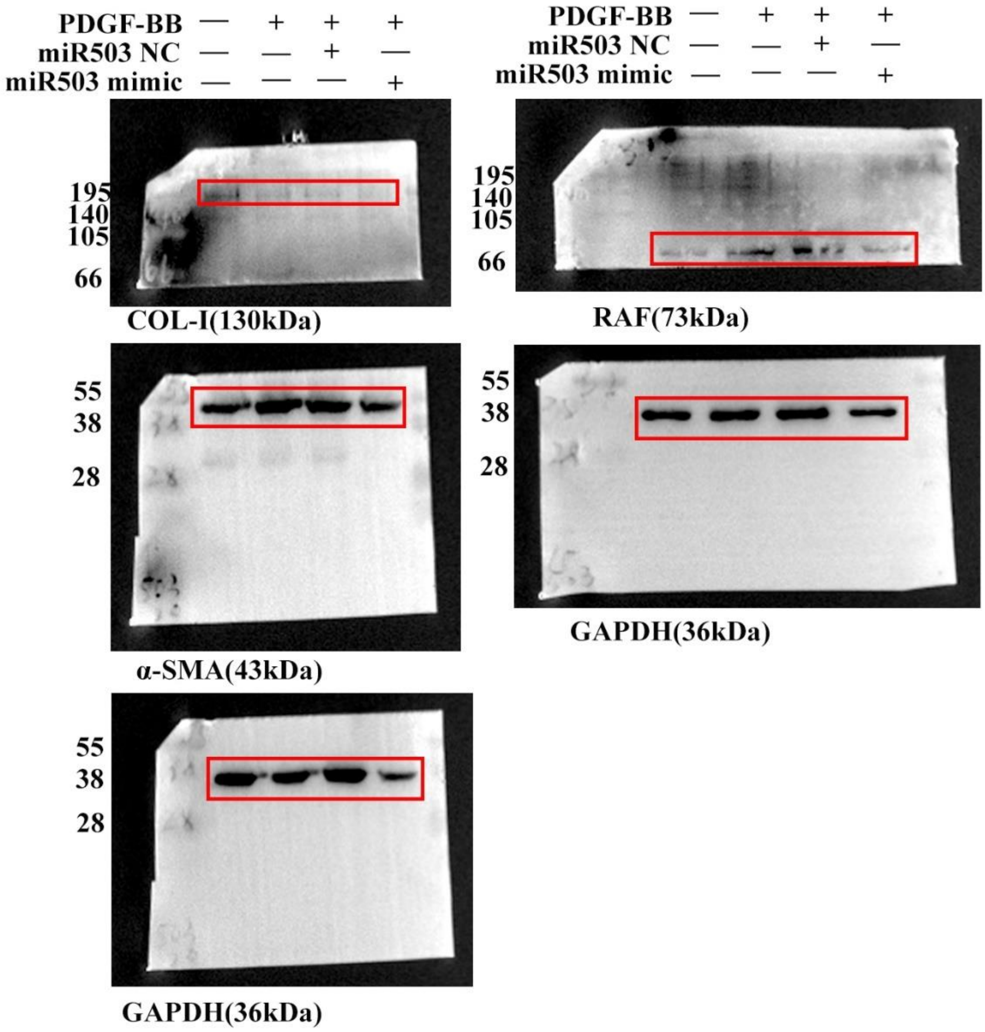

Repeat 3

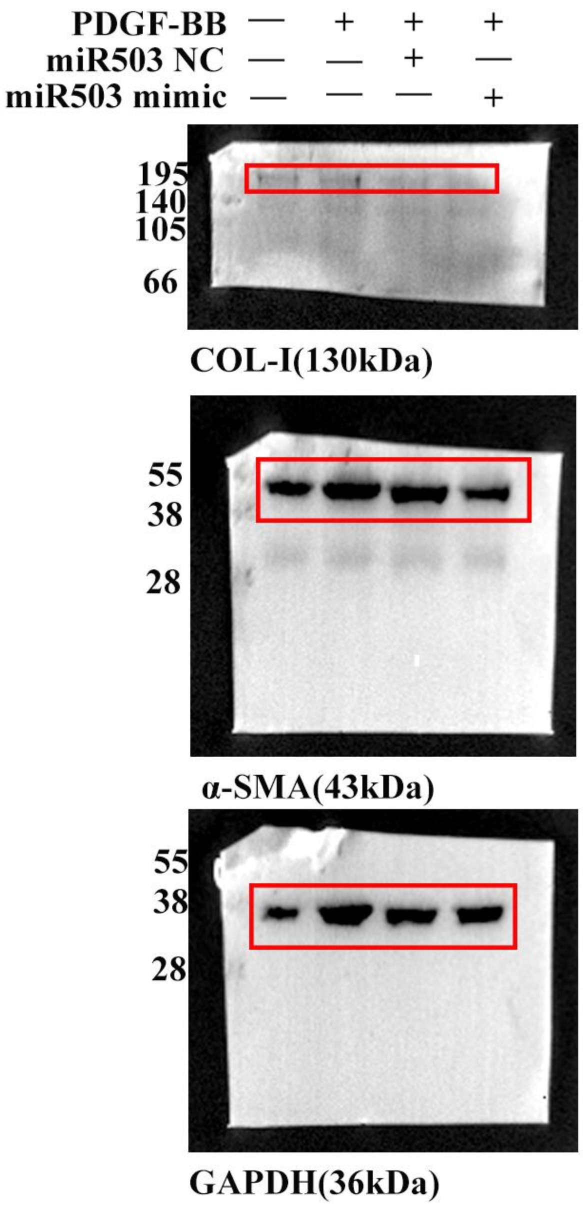
PDGF-BB — + + +
miR503 NC — — + —
miR503 mimic — — — +
195
140
105
66
COL-I(130kDa)
55
38
28
α-SMA(43kDa)
55
38
28
GAPDH(36kDa)

Figure3-5 A. Original western blot

Repeat 1

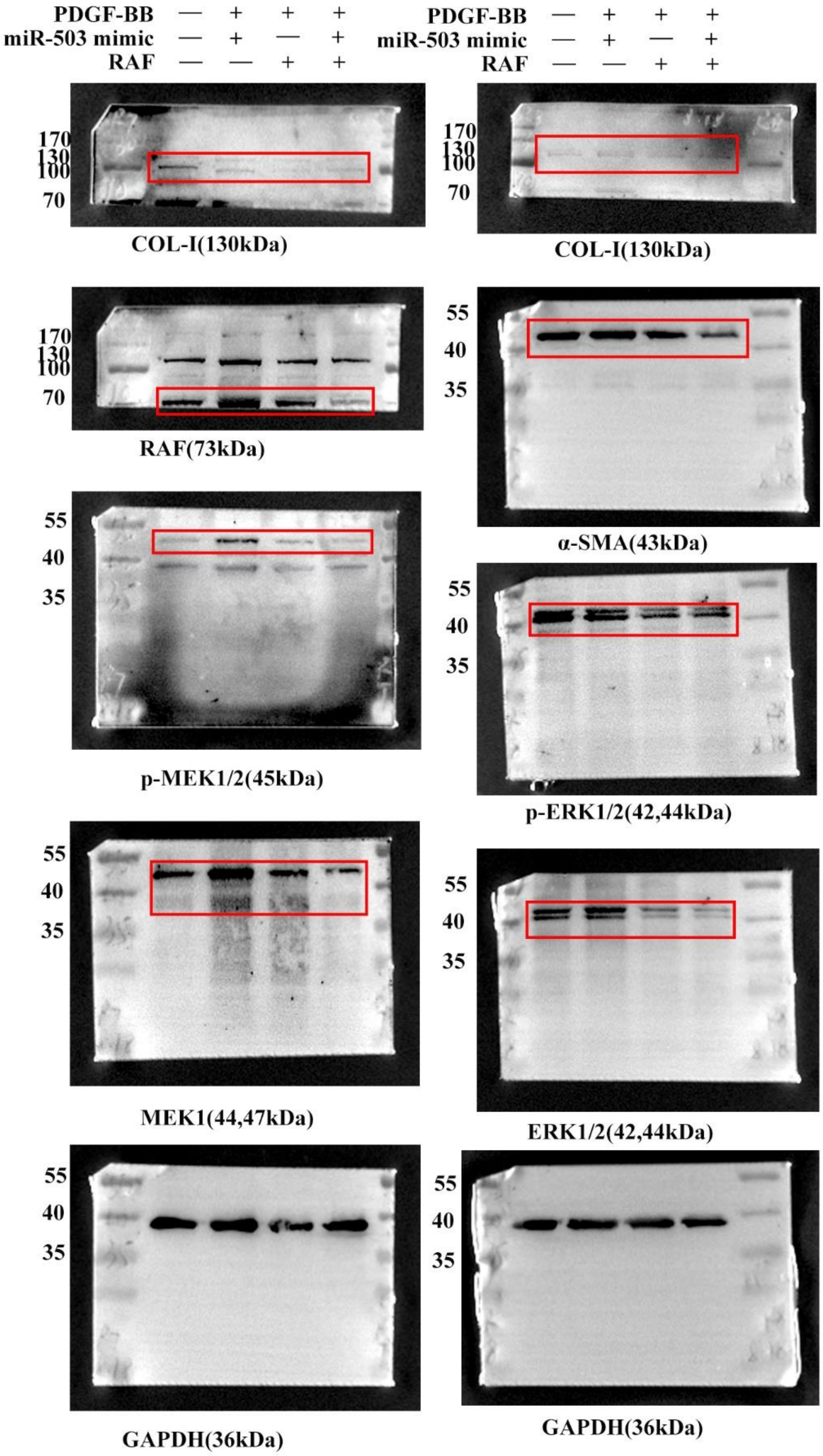

Repeat 2

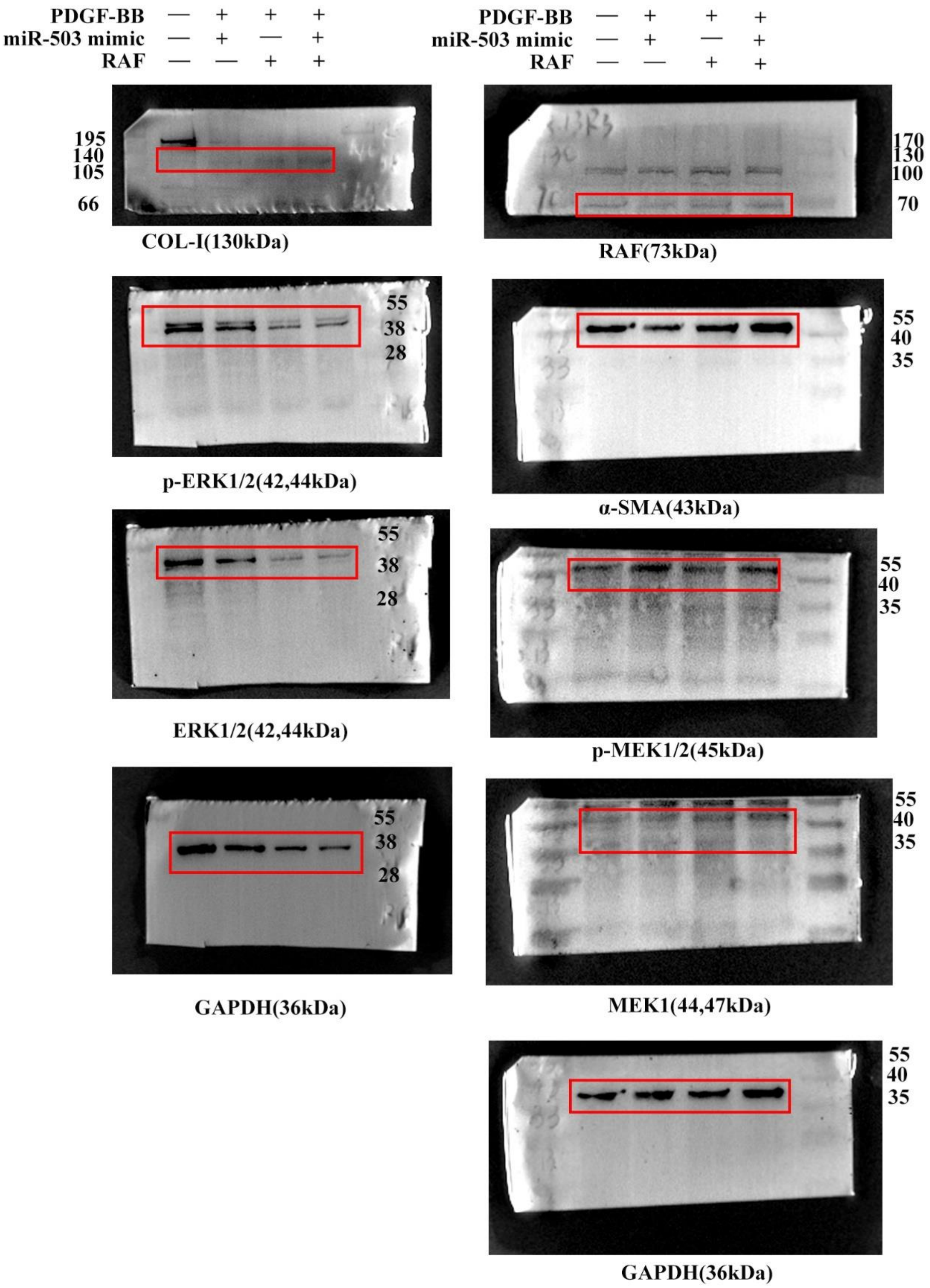
PDGF-BB — + + +
miR-503 mimic — + — +
RAF — — + +
PDGF-BB — + + +
miR-503 mimic — + — +
RAF — — + +
195
140
105
66
COL-I(130kDa)
170
130
100
70
RAF(73kDa)
55
38
28
p-ERK1/2(42,44kDa)
55
40
35
α-SMA(43kDa)
55
38
28
ERK1/2(42,44kDa)
55
40
35
p-MEK1/2(45kDa)
55
38
28
GAPDH(36kDa)
55
40
35
MEK1(44,47kDa)
55
40
35
GAPDH(36kDa)

Repeat 3

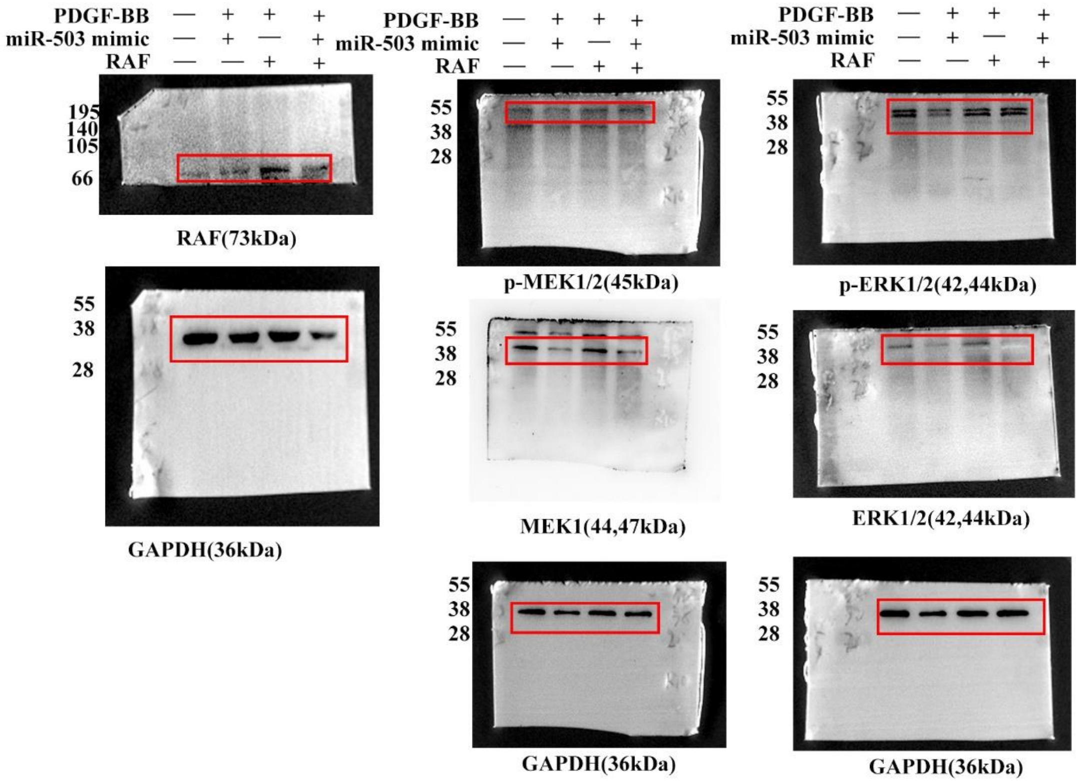
PDGF-BB — + + +
miR-503 mimic — + — +
RAF — — + +
195
140
105
66
RAF(73kDa)
55
38
28
GAPDH(36kDa)
PDGF-BB — + + +
miR-503 mimic — + — +
RAF — — + +
55
38
28
p-MEK1/2(45kDa)
55
38
28
MEK1(44,47kDa)
55
38
28
GAPDH(36kDa)
PDGF-BB — + + +
miR-503 mimic — + — +
RAF — — + +
55
38
28
p-ERK1/2(42,44kDa)
55
38
28
ERK1/2(42,44kDa)
55
38
28
GAPDH(36kDa)
